# Application of Empirical Wave Run-Up Formulas to the Polish Baltic Sea Coast

**DOI:** 10.1371/journal.pone.0105437

**Published:** 2014-08-19

**Authors:** Dominik Paprotny, Paweł Andrzejewski, Paweł Terefenko, Kazimierz Furmańczyk

**Affiliations:** Faculty of Geosciences, University of Szczecin, Szczecin, Poland; University of Vigo, Spain

## Abstract

Advanced, multidimensional models are typically applied when researching processes occurring in the nearshore. Relatively simple, empirical equations are commonly used in coastal engineering practice in order to estimate extreme wave run-up on beaches and coastal structures. However, they were mostly calibrated to the characteristics of oceanic coasts, which have different wave regime than a semi-enclosed basin like the Baltic Sea. In this paper we apply the formulas to the Polish Baltic Sea coast. The equations were adjusted to match local conditions in two test sites in Międzyzdroje and Dziwnówek, where beaches are under continuous video surveillance. Data from WAM wave model and coastal gauge stations were used, as well as precise measurements of the beaches' cross-sections. More than 600 run-up events spanning from June to December 2013 were analysed, including surges causing dune erosion. Extreme wave run-up *R_2%_* was calculated and presented as a percentage value indicating what part of the beach was inundated. The method had a root-mean-square error of 6.1 and 6.5 percentage points depending on the test site. We consider it is a fast and computationally undemanding alternative to morphodynamic models. It will constitute a part of the *SatBałtyk Operating System-Shores*, delivering forecasts of wave run-up on the beaches for the entire Polish coastline.

## Introduction

Coastal zone represents a dynamic environment where land, sea and atmosphere meet. It is also a system where up to two-thirds of the world population lives and many other are visiting those areas frequently [1], [2]. In order to sustainably manage recreational, industrial, agricultural and any other activities taking place near the shores it is necessary to understand the dynamic nature of both marine and terrestrial interactions at the coasts.

Small-scale elements of a depositional coast can experience rapid change in their morphology, mainly due to short-term sea level variations caused by storm surges [3]–[6]. In this perspective it is crucial for coastal managers to determine the impact of the sea on the coast especially in the aspect of potential hazard and risk to human activities as well as to the shore itself. This includes also the possibility of making predictions of those impacts.

Recently, an evaluation of risks, existing coastal management plans and civil engineering-produced protection schemes in nine European countries revealed that operational approaches to coastal risks and hazards had been virtually non-existent in most of those countries [7]. The biggest effort used to be channelled only to forecasting physical parameters (wind, waves, sea levels etc.), without considering their impact on the coast. One of the first steps to adopt an alternate approach to coastal hazards was the MICORE project in which nine prototype early warning systems based on open-source morphological XBeach model [8] were developed [9], [10]. MICORE made it possible to test, with good results, the XBeach model at a variety of test sites along the European coasts, including the Polish southern Baltic coast [11].

Advanced, multidimensional models such as XBeach are typically applied when researching processes occurring in the nearshore. They mathematically explain typically complex and non-linear relationships between different variables and are indispensable in enhancing knowledge about coastal systems [12], [13]. Models provide support in finding the best means to deal with as yet unforeseen events and their effects, both morphological and socio-economic. In Poland, efforts are being made to expand research on other parts of the coast. Currently three test sites are included in the *SatBałtyk Operating System-Shores* [14], the coastal-research component of a research project dedicated to the monitoring of the Baltic Sea [15]. Unfortunately, these models have a major drawback: they are computationally demanding, limiting the possibility to use them for detailed analysis of large parts of the shoreline.

But alongside those robust models some simpler methods were developed. At times when solving complex, non-linear equations was problematic to say the least, coastal engineers created empirical formulas. They were intended to improve the designs of coast protection structures. Wave run-up could be calculated and therefore enabled the engineers to design proper heights of embankments or seawalls [16], [17]. In the 1980s scientists started field experiments trying to apply the equations to natural beaches, an attempt that proofed successful. Several new equations were also developed [18]–[24]. Yet unlike more sophisticated methods, they are not being used in operational forecasting, but are rather confined to engineering practice.

Nevertheless, the reliability of aforementioned methods, both the empirical equations and models, needs always to be verified when applied to a new area. One possibility is to conduct *in situ* measurements of the desired phenomena. Much more convenient, though, is to use an increasingly popular alternative in the form of video monitoring systems. This method was applied for coastal research in the early 1980s and developed ever since. Video cameras were used during several experiments, capturing wave run-ups or measuring sand bar movements [25], [26]. Whereas traditional methods (*in situ* measurements, aerial and satellite imagery) are expensive and limited by weather conditions, video systems enable the researches to continuously monitor beach processes in the long-term [27], [28]. Especially since the technique has been automated and turned from analogue to digital, it has become a standard procedure when calibrating models for wave run-up and rip current occurrence [23], [27], [29].

Research described in this paper was conceived while examining possibilities to expand the beach inundation module of *SatBałtyk Operating System-Shores* beyond the three test sites it currently includes. Preliminary analysis found that running XBeach model (1D variant) for an estimated 500 cross-sections of the coast (one for each kilometre of the coast) would be too time-consuming and therefore render the resulting forecasts useless. Moreover, it was designed specifically sandy coasts, which is not necessarily the case along the Baltic coast. Empirical equations of wave run-up on slopes were chosen as an alternative, for several reasons. Firstly, they are computationally undemanding, therefore creating a possibility to deliver forecasts without substantial delays. Secondly, they utilize the very same data as the system based on XBeach: wave parameters from WAM model, sea levels from M3D model and Maritime Offices' cross-sections of the shore.

It excepted that the formulas, once calibrated, will be built into an operational, forecasting system during 2014–2015 in support of the *SatBałtyk* project [14]. Each 1 km cross-section will contain data extracted from the latest available high-resolution digital elevation models, created using lidar technology and provided by the responsible maritime offices.

Additionally, *Satbałtyk* project expects its system and models to be used, apart from delivering forecasts covering up to several days, also to archive the results and calculate hindcasts. They will allow us to conduct seasonal and long-term analysis. Therefore, alongside the calibration of the method, we include elements of such an investigation for two test sites, on which this experiment is based.

## Materials and Methods

Wave run-up is defined as “the landward extent of wave uprush measured vertically from the still water level” [24]. The earliest formulation of this is by Hunt [30], who gives the following equation:
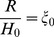
(1)where *R* is the wave run-up, *H_0_* is the deep-water wave height and *ξ_0_* is the deep-water surf similarity parameter. The latter is given as follows [31]:
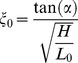
(2)where *α* is the slope angle and *L_0_* is the linear theory deep-water wave length:
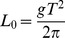
(3)where *T* is regular wave period. Hunt's formula can be used to calculate run-up from incidental regular waves. Later research reworked eq. (1) to include irregular waves and introduced additional empirical parameters based on field measurements and lab experiments [24]. Several very similar equations were developed, of which the most recent version from Mase [32] was chosen:
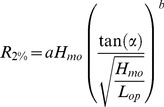
(4)where *R*_2%_ is wave run-up with a 2% probability of occurrence (i.e. 1 in 50 waves), *H*_mo_ is the significant deep-water wave height and *L*_op_ is the same as in eq. (3), except that *T* stands here for peak wave period (*T*_p_). (The 2% run-up is commonly used in similar equations, though the reason for such a choice is not well explained. It seems to have a special statistical relation to significant wave height and originate from Dutch experiments carried out in the 1930s, but the report where these results were published apparently has gone missing [17]). Additionally, two empirical parameters can be found in eq. (4): *a* and *b*. Mase suggested *a* = 1.86 and *b* = 0.71, based on his laboratory tests [32]. Several field tests have been conducted by various authors, but they were mostly located on high-wave-energy coasts of the United States, the Netherlands, Portugal, Spain or Australia [23], [28], [33], [34]. One study for the Baltic sea could be identified, to our best knowledge, albeit it did not include any adjustments of parameters [35]. As a result, the formula had to be calibrated on the Polish coast to ensure its accuracy. It should be also noted that other field-tested equations exist, developed by Hughes [22] and Stockdon [23]. Melby [24] tested those equations versus two incarnations of Hunt's formula using Stockdon's compilation of field experiments and found that Holman's [19] and Mase's equations had better statistical performance. Equation by Holman differs from Mase's only by the inclusion of a third parameter. Their performance, when calibrated, is virtually of the same quality, therefore Mase's variant was chosen as having less parameters to calibrate. This is even in spite of the fact that the main assumption behind the formula—a plain, impermeable slope—conforms more to flood protection structures than beaches, especially sandy ones.

**Figure 1:**
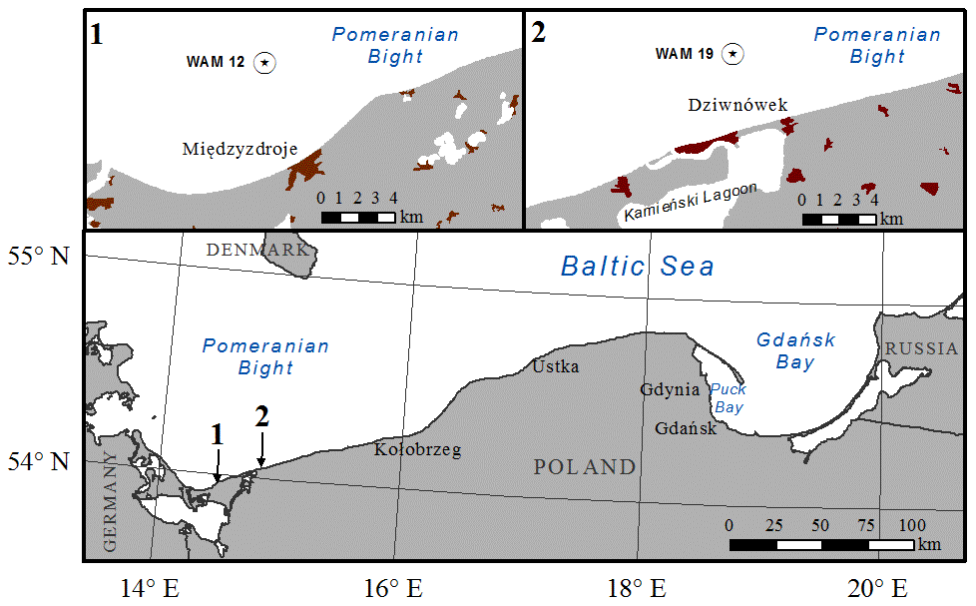
Polish Baltic Sea coast. Location of the test sites in Międzyzdroje (1) and Dziwnówek (2) is marked.

Polish Baltic Sea coast is 500 km long, including 72 km along the Hel Peninsula. Approximately 80% is a dune coast, whereas the remaining part forms a cliff coast [36]. Vast majority of the coastline has a beach, except for river mouths, harbours, military bases and the swampy shores of Puck Bay. Research was carried out in two locations in the western part of the coast, namely Dziwnówek and Międzyzdroje (Fig. 1).

Międzyzdroje is a town on Wolin Island, while Dziwnówek is a village located 30 km east of the former. Both are very popular summer resorts along the banks of the Pomeranian Bight. The bight is a shallow basin, where depths are generally below 20 meters. The coast in this area is oscillating, but in overall erosion is stronger than accumulation. It includes both cliffs and dunes, which are retreating, on average, by 10 cm per year [37].

The two selected cross-sections are located at about 414.2 and 386.2 km of the Maritime Office's kilometrage. Both shores include moderately developed dunes that reach 5–7 m above sea level. There are no coastal protection structures in those particular locations apart from some timber groynes in Dziwnówek. They are also under continuous video surveillance.

**Figure 2:**
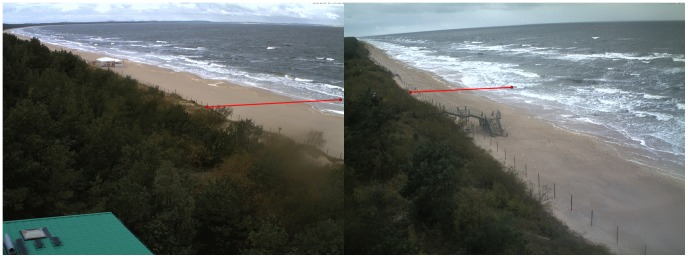
View from cameras in Międzyzdroje (left) and Dziwnówek (right). Cross-sections are marked in red.

In Dziwnówek a video camera was mounted in 2009 in order to monitor a test site for the MICORE project [7]. Another camera was installed in Międzyzdroje in 2013 to support model validation activities in the SatBałtyk project [14]. They are both placed at the tops of observation towers used formerly by the military and border guards. The view from the cameras is presented in Fig. 2 (real-time images could also be found online [38]). The cameras were specifically adjusted for use as a close-range remote sensing device through a calibration processes described in literature [39], [40].

The cross-sections under observation were surveyed with GPS-RTK devices while the cameras were operational, so that the exact location of each point along the profile could be unequivocally identified on all images from video monitoring. Measurements were conducted on July 11^th^, 2013 (Dziwnówek) and July 18^th^, 2013 (Międzyzdroje). In Dziwnówek the length of the profile, measured from the waterline at mean sea level to the base of the dune, is almost 33 m with an elevation difference of 2.32 m. For Międzyzdroje the values are 53 m and 1.88 m, respectively. Detailed data can be found in Table S1A in File S1.

**Figure 3:**
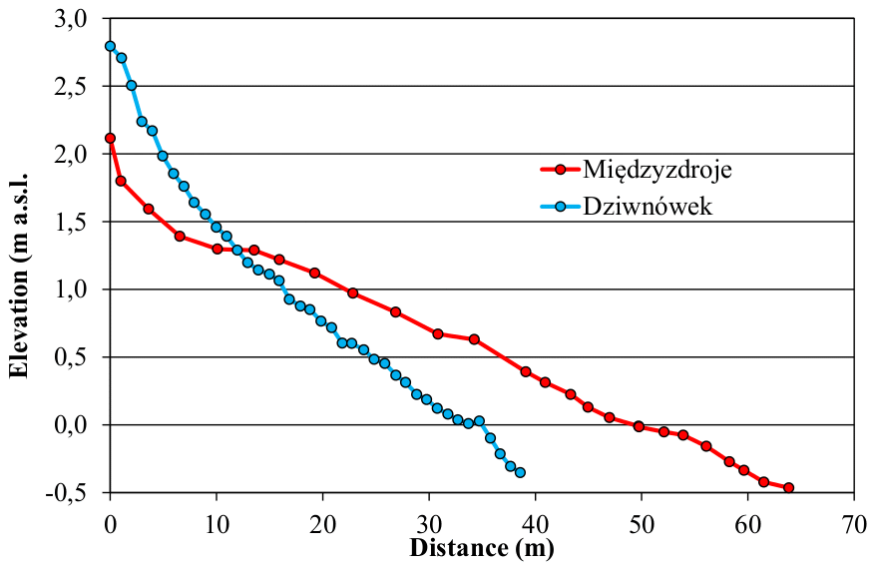
Cross-sections of two beaches in Międzyzdroje and Dziwnówek.

A major issue is calculating the slope angle properly. The method assumes a single value, while in reality the beaches are more complex. The profiles shown in Fig. 3 are relatively smooth and do not include a longshore bar that typically appears near the waterline after storms. Presence of a sand bar can skew the results, but it did not appear throughout the course of the experiment. Due to the limitations of the measurement method used, the cross-sections do not include most of the underwater part of the foreshore. Approaches to the slope angle problem differ largely. For example, Douglass [20] suggested skipping the slope parameter altogether, based on his field experiments on the US Atlantic coast. On the opposite end, an ‘effective slope’ method was presented as the most accurate by Mayer and Kriebel [41], albeit it is most cumbersome to apply, as the slope angle is recalculated for every event between the wave breaking point and run-up on the slope (an iterative method). For the purpose of this study, the angle was calculated using linear regression based on all available measurements from the underwater part up to and including the dune base. Consequently, the angle amounts to 3.9° in Dziwnówek and 2.0° in Międzyzdroje. Notwithstanding, large disproportion between beach height and width (1∶12 and 1∶23, respectively) means that the assumption of a plain slope is still fairly accurate, at least as long the beach is not reshaped by a storm.

The final element of eq. (4) that had been acquired were data on waves, i.e. deep-water significant wave height and peak wave period. Since no actual measurements from buoys were available when the research described here was carried out, calculations from WAM wave model were used instead. This two-dimensional model of wave spectrum [42] was operationally implemented for the Baltic Sea by the Interdisciplinary Centre for Mathematical and Computational Modelling [43]. It provides a 84-hour forecasts every 12 hours [44] (ICM 2014). The latest forecast for each event included in the calibration process was used. ICM's model has a 8 km grid; central points of those grids utilized in this study are marked in Fig. 1. WAM point no. 12 supplied information for Międzyzdroje, while point no. 19 for Dziwnówek.

One additional factor had to be included, namely sea level, because eq. (4) calculates run-up from still water level. Measurements were obtained from the Institute of Meteorology and Water Management, which publishes real-time data from tide gauges on its website [45]. Levels from gauges in Dziwnów and Świnoujście were used for the calibration process in Dziwnówek and Międzyzdroje, respectively. The Baltic is a non-tidal basin, which also marks a difference from other research, which was done hitherto on tidal coasts.

Wave run-up was extracted from two time periods during every day from June 20^th^ up to December 8^th^, 2013. Such approach differs from other experiments [23], which analysed a few days or weeks in great detail. Here, almost half a year in less detail was chosen in order to ensure the method's validity over a longer period of time, given our ultimate goal of creating a forecasting tool. Only days with considerable wave heights or sea levels were analysed in one-hour periods, which was the temporal resolution of hydrologic data available. Such events occurred on 23^th^–24^th^ of September, 24^th^–26^th^ and 28^th^–29^th^ of November, 2^nd^ and 5^th^–7^th^ of December. This last event was a fierce winter storm *Xavier*, which completely reshaped the beaches, creating a vast sandbar near the waterline and an indention towards the middle of the profile. In effect, calibration process was not continued after that date. During periods of calmer weather, wave run-ups from around 7 and 13 UTC were an analysed, with small alterations on some days due to availability of data. Thanks to this approach, small inundations of 0–20% would not completely dominate the calculations, even though they still represented the bulk of data acquired.

For each event that was selected, up to 15 minutes of footage were observed in order to extract the highest wave run-up on all images. Since there would be about 100 individual wave run-ups during this period of time the value obtained from the images is likely to correspond to the 2% run-up modelled by eq. 4 [33], [46]. Measurements of the highest run-up during in the 15-min set of images sampled at 1 Hz were made along the profile lines mentioned above. Distance between the point of the highest run-up and the ‘zero’ point could therefore be obtained from the image. Thanks to GPS RTK measurements at different points along the profile line, the distance in pixels could be easily transformed to distance in the terrain. As a result, a percentage value of beach inundation could be obtained. The ‘zero’ point (0% extreme wave run-up) was located at the point of the profile that equals the mean sea level, defined as −0.08 m in Kronstadt-86 reference system or 500 cm at Polish gauge stations. On the other end of the scale, 100% run-up would mean that the extreme (2%) waves reached the dune base. The distance between those points constitutes the ‘baseline’ beach width. Therefore, modelled extreme beach inundation *B* could be written as:
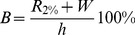
(5)where *R*_2%_ is the wave run-up from eq. (4), *W* is the sea level and *h* is the ‘baseline’ height of the beach. The inundation parameter could a have a negative value, since the sea level could be so low, that the waves reached below the mean sea level point. Therefore, a −10% value indicates that the beach is at the moment 10% longer than the ‘baseline’ width. On the other hand, maximum values of *B* were capped at 100%, since any higher value means that the waves reach outside the beach.

**Figure 4:**
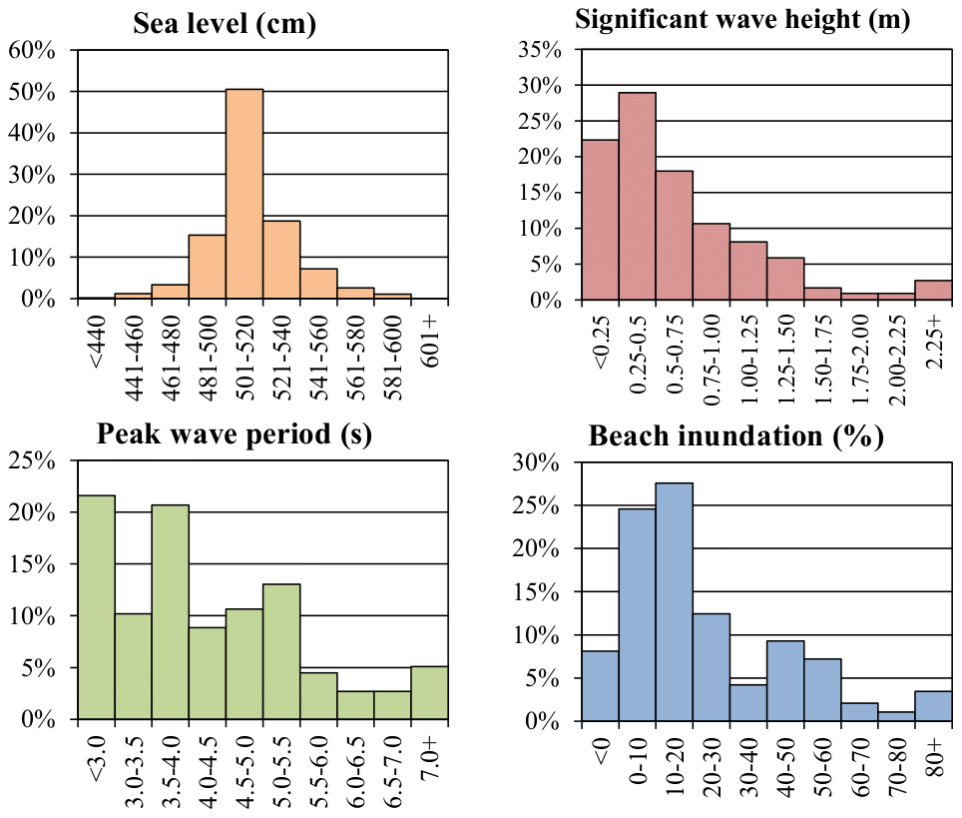
Histograms of parameters included in the study. Beach inundation pertains to actual (measured) wave run-up as % of the beaches' baseline width.

A total of 667 events were analysed: 367 in Międzyzdroje and 300 in Dziwnówek (the latter number is lower due to camera malfunctions). During those events, water levels varied from 426 cm to 596 cm; significant wave heights reached from 0.01 to 3.99 m and peak wave periods from 2.0 to 10.2 seconds. Large variety of values indicate that both storm surges and falls were included, as well as period of calm weather. The lowest measured beach inundation amounted to −22.5% in Międzyzdroje and −7.3% in Dziwnówek, while during the *Xavier* storm waves swashed the dunes in both locations (100% run-up). Histograms are presented in Fig. 4. Underlying data (except for sea levels) can be found in Table S1B in File S1.

Having obtained actual wave run-ups, empirical parameters *a* and *b* where adjusted, so that the linear regression line plotted using the data for each locality would pass through points of equal value of both actual and modelled run-up, i.e. constitute a 1∶1 line. The correlation between the modelled and actual data was not maximized here, but it decreased the formula's bias in estimating high run-ups.

A permit was required in order to conduct GPS measurements on the dunes. It was obtained from the Maritime Office in Szczecin. No other permissions were necessary. Video cameras used in this study were placed on the university's (Międzyzdroje) or private (Dziwnówek) parcels by kind consent of their respective owners.

## Results

**Figure 5:**
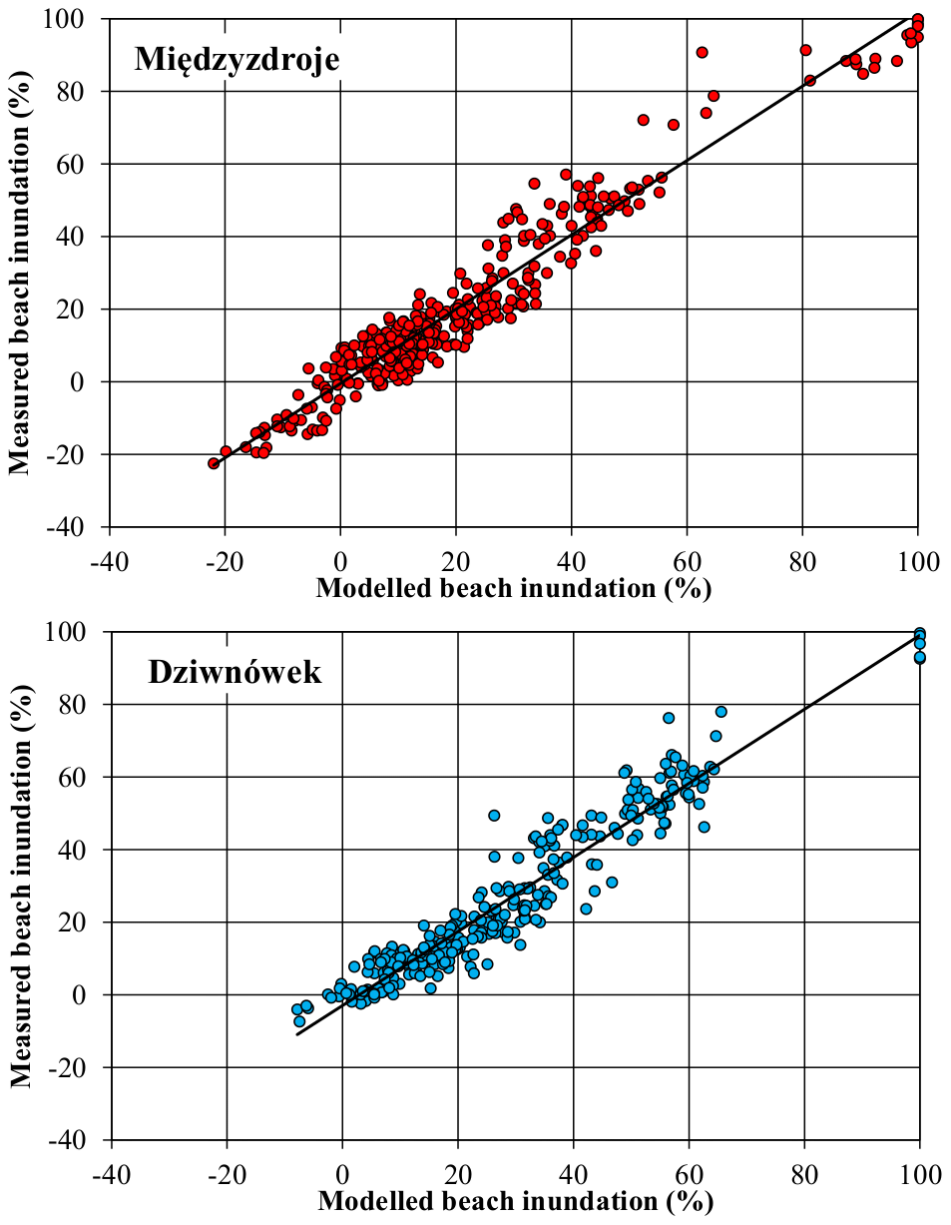
Measured and modelled beach inundation for Międzyzdroje and Dziwnówek.

Results of the calibration process are presented in Fig. 5. Empirical parameters *a* = 1.29 and *b* = 0.72 where used, so that eq. (4) becomes:
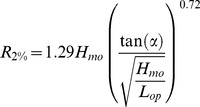
(6)


In general, the data concentrate along the regression line, with very few outlying elements. Root-mean-square error for data from Międzyzdroje is 6.1 percentage points, or 3.2 m of beach width. In Dziwnówek, the error is slightly larger, 6.5 percentage points, but because the beach is shorter, this value corresponds to 2.2 m of its width. The coefficient of determination is high and amounts to 0.94 in Międzyzdroje and 0.92 in Dziwnówek.

In Międzyzdroje, the formula proved most accurate for small run-ups, up to 30%, while more energetic events where slightly underestimated. In Dziwnówek, it is the opposite. However, these are only slight deviations from the line of equality. On most occasions. Unfortunately, only a few cases of 60–80% run-ups where recorded, causing some uncertainty about the method's reliability for storm events, even though ca. 90% beach inundation was estimated correctly. It generally properly predicted waves reaching the dune base.

However, the impression of very good quality of the model is slightly misleading. An important factor regulating how far will the wave swash into the beach is the sea level. It contributed, on average, to about 22% of the actual beach inundation in Międzyzdroje and 32% in Dziwnówek. Since the data from tide gauges are fairly accurate (even though there is some considerable distance from the gauge station to Międzyzdroje), performance of ‘bare’ wave run-ups was calculated. Normalized RMS error amounted to 0.27 in Międzyzdroje (i. e. average error in estimating wave run-up was 27%) and 0.29 in Dziwnówek. Coefficient of determination was 0.85 and 0.81, respectively. This is a lower value than that recorded by the beach inundation indicator, but still a decent result.

**Figure 6:**
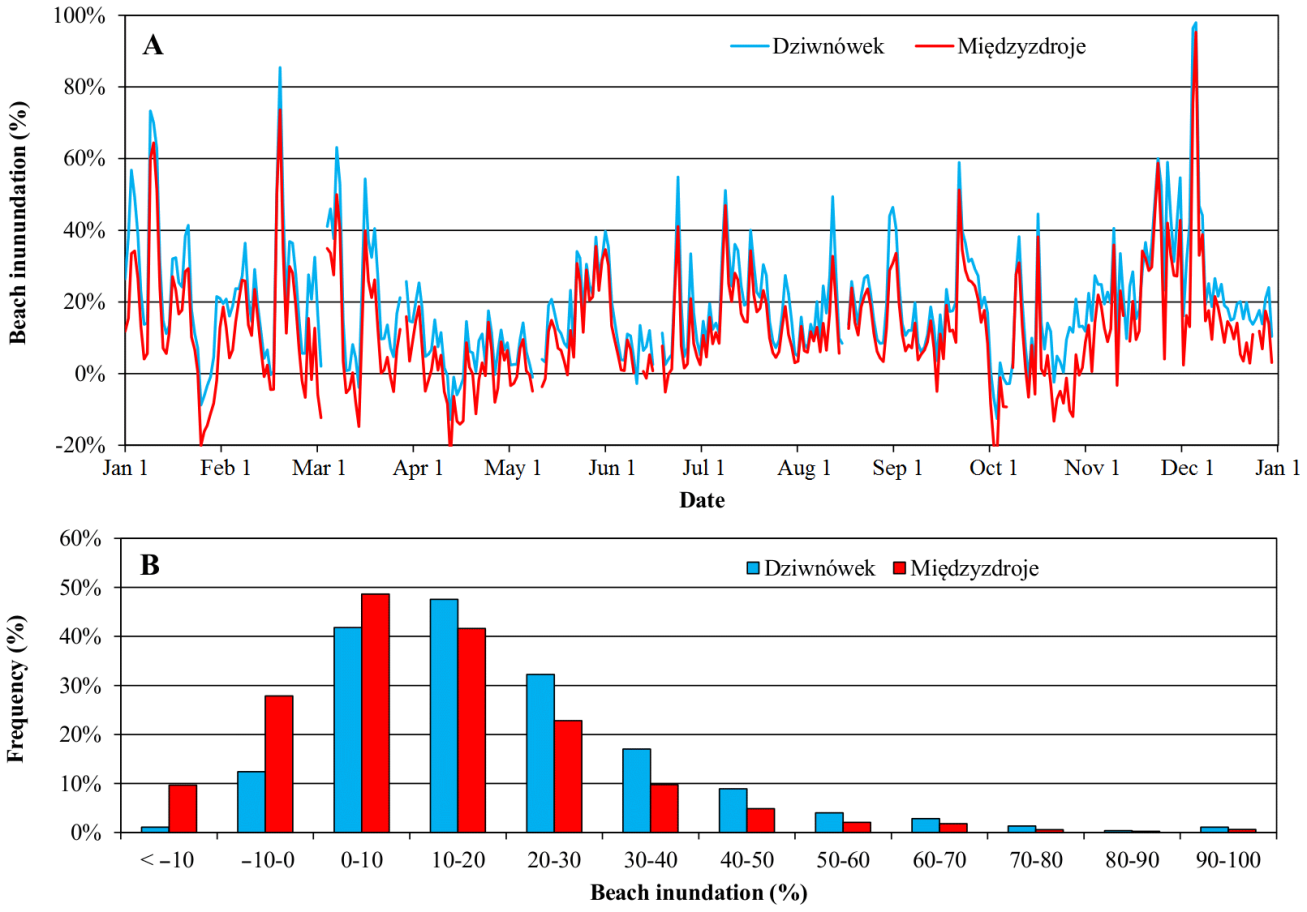
Extreme 2% beach inundation estimates for Międzyzdroje and Dziwnówek, hourly data from January 1^st^ to December 31^st^, 2013. Daily averages (A) and histogram (B).

Having obtained optimal parameters for the formula, wave run-up was recalculated using hourly data from WAM and M3D models. The latter is a hydrodynamic model providing, among other parameters, water levels for the Polish coast [47], [48]. Fig. 6 presents a histogram of inundation from hourly data and its change over the course of 2013 (daily averages are presented for clarity of the graph). Run-ups below 30% constituted four-fifths of all events. The values for the beach in Dziwnówek were generally higher than in Międzyzdroje (20% against 12%). The reasons could be easily identified: not only Dziwnówek has a shorter and steeper beach, but also had higher waves (*H_mo_* = 0.53 m against 0.47 m) and water levels (504 cm against 500 cm) during that period. The beach in Międzyzdroje was more inundated than Dziwnówek's only 5% of the time.

In Fig. 6 both calmer and more dynamic periods are visible. Especially standing out is the *Xavier* storm surge in early December. Only one event in February comes close. November and December observed the highest monthly means, with April and October on the opposite. In Dziwnówek, 18 days had average with inundation above 50%, whereas in Międzyzdroje the corresponding figure is 9 days. 2013 was not a particularly eventful year in terms of storms in general. Even *Xavier*, though generated strong winds and caused damages in some Western countries was at the end rather unimpressive in terms of coastal flooding. It could be estimated that it impacted the dunes in Dziwnówek for around one and a half day, and even less than that in Międzyzdroje. Naturally, the results for parts of the year could be not exactly accurate due to changes in morphology of the beaches, particularly after the December surges, where sandbars near the waterline appeared.

## Discussion and Conclusions

The empirical parameters' values from eq. 6 do not differ much from other authors' findings. Parameter *b*, estimated at 0.72, is almost the same as Mase's lab-tests result of 0.71 [32] and Melby's approximation of 0.70 based on Stockdon's dataset of nine different field experiments [23], [24]. Such a small difference between different studies could indicate that the value of around 0.7 has a universal application. On the other hand, *a* parameter's value (1.29) is significantly lower than Mase's 1.86 [32]. Other authors' estimates of this parameter ranged from 1.49 to 1.87 [24]. It is likely caused by a different research scope: Mase and others tested their methods using very large slope angles that are typical for hydrotechnic structures such as dykes. In contrast, Melby's estimate from field data is 1.10 [24]. It is likely caused by different environmental conditions, since the beaches used in that study were located along tidal coasts with high wave energies. Another source of the difference may be the permeability of the beaches, though authors working on the empirical equations generally did not give much consideration to this aspect apart from assuming that the slopes they tested were impermeable or permeable, therefore is hard to quantify the impact of this factor. Overall, bigger permeability lowers run-up [49] and the Polish beaches are definitely more permeable than those used in other studies.

Our results could be compared with other findings using Melby's Summary Performance Score. This statistic is an average of normalized RMS error, normalized bias and scatter index with an 0 to 1 scale, where 1 means no error [24]. Only wave run-up from still water level is taken into account here. In our case, the Performance score amounted to 0.78 in both locations. Application of Mase's equation adjusted by Melby to Stockdon's field data gave a result from 0.62 to 0.90 depending on the test site. Four out of nine beach had better score than 0.8, with an average of 0.84 [24]. Melby's findings were more accurate than our results, though it should be remembered that they were based on a much smaller number of run-up events (from 14 to 138, depending on the test site). Additional error could have come from the limitations of our dataset: the use of modelled wave data and inaccuracies in registering wave run-ups from images. Nevertheless, it can be concluded that the results of the calibration process were in line with other authors' findings.

Notwithstanding, some shortcomings of the method could be identified in the context of application for the whole Polish coast in *SatBałtyk Operational System-Shores*. First of all, the two test sites are both located on the western part of the coast, therefore not necessarily accurate enough for the middle and eastern section. Currently there is no possibility of validating the model there, since no suitable, scientific cameras are installed along the coast. What is more, the method has its potential inaccuracies originating in the heavy reliance on other models' output: modelled wave data are used for both calibration and operational forecasting. All this means that the method described here, though accurate for the test sites, should be subjected to further validation in other areas, before implemented to the entire Polish coast in an operational system.

When completed, the forecasting system could have several applications. For example, temporary constructions that are placed on the beach in the summertime could be removed in time to protect them from the effects of high wave run-up. Moreover, run-up predictions close to 100% would indicate potential dune/cliff erosion, an information useful for scientists interested in the subject of coastline change. As could be seen in Fig. 6 the method is also useful for analysing storm surges' development over time, as well as seasonal analysis. The topic will be investigated further in the future. More work is also expected on the calibration, too, pending availability of new data.

To sum up, the study proved that empirical wave run-up formulas could be applied to the Baltic Sea after calibration to local conditions. The method is accurate enough to be used in other work, especially lays foundations for an operational, forecasting system (which will naturally depend also on the quality of operational hydrologic models delivering data for calculating beach inundation). The errors recorded were not substantial despite applying a formula designed for very different conditions. This proves that this simple method, if properly used, can also be a valuable addition to more complex research.

## Supporting Information

File S1 Contains: Table S1A. Cross-sections of the beaches. Table S1B. Extreme wave run-ups and wave data.
(XLS)
